# Cytokeratin 15 Marks Basal Epithelia in Developing Ureters and Is Upregulated in a Subset of Urothelial Cell Carcinomas

**DOI:** 10.1371/journal.pone.0081167

**Published:** 2013-11-18

**Authors:** Guangping Tai, Parisa Ranjzad, Fiona Marriage, Samrina Rehman, Helen Denley, Jill Dixon, Karen Mitchell, Philip J. R. Day, Adrian S. Woolf

**Affiliations:** 1 Faculty of Medical and Human Sciences, University of Manchester, Manchester, United Kingdom; 2 Faculty of Life Sciences, University of Manchester, Manchester, United Kingdom; 3 Manchester Centre for Integrative Systems Biology, University of Manchester, Manchester, United Kingdom; 4 School of Chemical Engineering and Analytical Science, University of Manchester, Manchester, United Kingdom; 5 Department of Histopathology, Manchester Royal Infirmary, Manchester, United Kingdom; National Cancer Institute, United States of America

## Abstract

The mammalian ureter contains a water-tight epithelium surrounded by smooth muscle. Key molecules have been defined which regulate ureteric bud initiation and drive the differentiation of ureteric mesenchyme into peristaltic smooth muscle. Less is known about mechanisms underlying the developmental patterning of the multilayered epithelium characterising the mature ureter. In skin, which also contains a multilayered epithelium, cytokeratin 15 (CK15), an acidic intermediate filament protein, marks cells whose progeny contribute to epidermal regeneration following wounding. Moreover, CK15+ precursor cells in skin can give rise to basal cell carcinomas. In the current study, using transcriptome microarrays of embryonic wild type mouse ureters, *Krt15*, coding for CK15, was detected. Quantitative polymerase chain reaction analyses confirmed the initial finding and demonstrated that *Krt15* levels increased during the fetal period when the ureteric epithelium becomes multilayered. CK15 protein was undetectable in the ureteric bud, the rudiment from which the ureter grows. Nevertheless, later in fetal development, CK15 was immunodetected in a subset of basal urothelial cells in the ureteric stalk. Superficial epithelial cells, including those positive for the differentiation marker uroplakin III, were CK15-. Transformation-related protein 63 (P63) has been implicated in epithelial differentiation in murine fetal urinary bladders. In wild type fetal ureters, CK15+ cells were positive for P63, and *p63* homozygous null mutant ureters lacked CK15+ cells. In these mutant ureters, sections of the urothelium were monolayered versus the uniform multilayering found in wild type littermates. Human urothelial cell carcinomas account for considerable morbidity and mortality. CK15 was upregulated in a subset of invasive ureteric and urinary bladder cancers. Thus, in ureter development, the absence of CK15 is associated with a structurally simplified urothelium whereas, postnatally, increased CK15 levels feature in malignant urothelial overgrowth. CK15 may be a novel marker for urinary tract epithelial precursor cells.

## Introduction

The mammalian ureter acts as a conduit, receiving urine from the kidney and propelling urine distally towards the urinary bladder. The mature ureter contains a multilayered epithelium, the urothelium, surrounded by smooth muscle. Several molecules have been defined which mediate the initiation of the ureteric rudiment, or ureteric bud (UB), and the differentiation of ureteric smooth muscle cells [1,2]. Instructed by paracrine signals, including glial cell line derived neurotrophic factor and fibroblast growth factors (FGFs) [3-5], the UB initiates from the mesonephric duct. The UB then grows into, and branches within, a zone of intermediate mesoderm called the renal mesenchyme to form the renal pelvis and collecting ducts of the metanephric kidney. The extra-renal section of the fetal ureteric stalk elongates and its primitive epithelium acts as a signalling centre, releasing sonic hedgehog which, acting with bone morphogenetic protein-4 and teashirt-3, induces nearby mesenchymal cells to differentiate into layers of smooth muscle. The latter begin to undergo peristalsis, coinciding with the onset of production of urine by the metanephric kidney [6,7]. 

The urothelial cells adjacent to the ureteric lumen are highly differentiated and these umbrella cells constitute a water-tight barrier, a property conferred by uroplakin protein heterodimers inserted into their apical plasma membranes [8,9]. In mice and humans, mutations of genes coding for uroplakin proteins lead to congenital renal tract malformations [10,11]. Less is known about mechanisms underlying the developmental patterning of the multilayered epithelium which characterises the mature ureter. 

In mammalian skin, another multilayered epithelium, the acidic intermediate filament protein cytokeratin 15 (CK15) is a marker for less differentiated, basal epithelia and for a subset of cells surrounding the hair follicle [12-16]. Genetic cell lineage studies in mice have provided evidence that the progeny of such CK15+ cells contribute to epithelial repair following wounding [15]. Moreover, at least in mice, CK15+ precursor skin cells can give rise to basal cell carcinomas [17]. 

In the current study, we explored CK15 in murine ureteric development and in human cancers arising from the urothelium. The results show that, during development, the appearance of CK15 coincides with the formation of a multilayered urothelium whereas, postnatally, increased CK15 levels sometimes feature in malignant urothelial overgrowth. 

## Materials and Methods

### Mice, human tissues and chemical reagents

Ethics Statement: Wild type CD1 mice and *transformation-related* protein *63* (*p63*) null mutant mice [18,19] were maintained in our local Biological Services Facility. Experiments were ethically-approved by the Registered Medical and Scientific Departments of the University of Manchester and the Home Office. The morning after mating was designated as embryonic day (E) 0. Mouse dissected tissues were frozen for RNA analyses, fixed in 4% paraformaldehyde prior to histology or used for organ culture experiments, as described below. Anonymised human tissue array slides for immunostaining were purchased from US Biomax Inc; their use does not require local investigator ethics approval. Reagents were obtained from Sigma Chemical (Poole, UK), unless otherwise stated.

### Embryonic mouse ureter organ culture

E14 ureters were explanted onto Millicell inserts (Millipore, Bedford, MA) and grown at 37°C in a humidified atmosphere of air/5% CO_2_. They were fed with defined, serum-free medium comprising DMEM/F12 (GIBCO BRL, Gaithersburg, MD), insulin (10 mg/l), sodium selenite (5 μg/l), and transferrin (5.5 mg/l), as described [7,20]. In some experiments, this control medium was supplemented with 100 ng/ml fibroblast growth factor 7 (FGF7; R&D Systems). Media was changed after three days. Explants were photographed as whole-mounts at days 0, 3 and on the final day of the 6-day culture period and their lengths measured by Axiovision software (Zeiss). In some experiments, 5-bromo-2'-deoxyuridine (BrdU; Roche), a synthetic thymidine analogue, was added two hours before the end of the experiment. Cultures were fixed in either 4% paraformaldhyde or ice cold methanol prior to whole mount imaging or histology. 

### Histology

Fixed mouse tissues were embedded in paraffin and sectioned at 5 μm. Paraffin tissue microarray slides of human ureters (UR1001) and urinary bladders (BL804) were purchased from US Biomax Inc. As assessed by a trained histopathologist (H.D.), a small subset samples in the arrays comprised only sparse tissues or had an apparently incorrect histological type cited in the data sheet. These samples were not analysed further. After dewaxing, slides were boiled in a microwave for 20 minutes in antigen retrieval solution (10 mM sodium citrate, pH 6.0). Sections were blocked with 1% bovine serum albumin and immunohistochemistry was undertaken using the following primary antibodies: rabbit anti α-smooth muscle actin (1:500, ab5694 Abcam), rabbit monoclonal anti CK15 (1:200, EPR1614Y, Abcam) (Sakomoto 2012), rabbit polyclonal anti CK19 (1:250; ab15463, Abcam), mouse anti E-cadherin (1:200; 610181, BD Biosciences), mouse anti P63 (1:500; 4A4, Abcam) which detect all P63 isoforms and goat anti uroplakin III (1:250; sc-15186, Santa Cruz Biotechnology). For brightfiled miscroscopy, primary antibodies were detected using appropriate secondary antibodies which were detected with a peroxidase-based method, as descibed [7,11], and some slides were counterstained with hematoxylin. Other sections probed with primary antibodies were then reacted species-specific fluorescent second antibodies (Molecular Probes) and slides mounted with Vectashield medium (Vector Labs) containing 4’, 6-diamidino-2-phenylindole (DAPI) to detect cell nuclei. Slides were viewed with an Olympus BX51 upright microscope. Images were captured using Cool snap ES camera (Photometrics) through MetaVue Software (Molecular Devices) or using a Nikon digital camera and analyzed using Image J, Paintshop software. Negative controls consisted of omission of primary antibodies and these experiments showed no significant signal (data not shown). To detect apoptotic nuclei, the Fluorescein In Situ Cell Death Detection Kit (Roche) was used for terminal deoxynucleotidyl transferase dUTP nick end labeling (TUNEL). In the *Results*, below, murine immunostaining patterns are depicted which were representative from three to five mouse organs in each experimental group, unless otherwise stated.

### RNA microarrays analyses

For microarray analyses, ureters were dissected from E14 and E16 wild type mouse embryos. Each ureter was divided in its longitudinal midpoint into a top (proximal) and a bottom (distal) half which were placed into Trizol lysis buffer (Invitrogen). Four gestationally- and spatially-equivalent half ureters were pooled to constitute a single ‘sample’ for each array. Four such samples were collected for each of the following groups: E14 top, E14 bottom, E16 top and E16 bottom. RNA was extracted using a combined Trizol (Life Technologies) and RNeasy kit (Qiagen) methodology and quantified prior to further analyses. GeneChip Mouse Genome 430 2.0 Array (Affymetrix) were used according to the manufacturer’s instructions [21]. The CEL files (ArrayExpress accession: E-MEXP-3929) were uploaded and analysed with the R-Bioconductor tools suite [22] using A) limma and B) puma [23]. Comparisons, with a focus on *Krt* transcripts/probe sets (Table 1) were carried out as follows: A) Limma (linear models for microarray data). Background correction and quantile normalisation were performed using RMA in R-Bioconductor. Principal component analysis on normalised data was implemented to test the quality of the array data in R to confirm correlation between clusters in related gene array chips. Differential expression analysis was accomplished with Limma using the functions lmFit and eBayes. The analysis was performed by creating design and contrast matrices for the following sample replicates: Top Ureter E16 versus Top Ureter E14, and Bottom Ureter E16 versus Bottom Ureter E14. Transcripts were selected and reported in terms of p-value and fold change from the mean were compared using the FDR q-value function in R. Results with a q-value 0.05 at the 95% confidence level were considered significant. B) Puma (propagating uncertainty in microarray analysis). Normalisation and expression analysis was done using multi-mgMOS. Differential expression between the sample groups was assessed with the puma package in R. Puma is a Bayesian method which includes probe-level measurement error when assessing statistical significance. Gross differences between arrays were determined using the puma variant of PCA; pumaPCA which can make use of the uncertainty in the expression levels determined by multi-mgMOS. Unlike many other methods, multi-mgMOS provides information about the expected uncertainty in the expression level, as well as a point estimate of the expression level. Differential expression (DE) analysis was performed with the pumaDE function. The results of these commands were ranked gene lists in order of probability of positive log-ratio (PPLR) values, and the FC values. *Krt* transcripts/probe sets were selected and reported in terms of PPLR values. PPLR values range from 0 to 1, with values closest to 0 representing the most significantly down-regulated probe sets and values closest to 1, representing the most significantly up-regulated probe sets. Values of 0.5 represent no significant change.

**Table 1:** *Krt* family transcripts in ureter development, as assessed by RNA arrays.

**Affy ID**	**Transcript Name**	**Bottom.16 vs. Bottom.14**			**Top.16 *vs*.Top.14**		
		pplr	p.value	q.value	pplr	p.value	q.value
1422481_at	Krt1	0.37963	0.4209	0.4452	0.11624	0.0249	0.0986
1427154_at	Krt2	0.50323	0.4619	0.4639	0.52771	0.0826	0.1844
1418735_at	*Krt4*	0.47142	0.8244	0.5969	*0.00100*	*0.0014*	*0.0206*
1438394_x_at	*Krt4*	0.53526	0.8580	0.6061	*0.00138*	*0.0008*	*0.0146*
1424096_at	Krt5	0.60885	0.0990	0.2081	0.39254	0.3884	0.3940
1422783_a_at	**Krt6a**	**1.00000**	**0.0005**	**0.0083**	**0.99985**	0.0199	0.0876
1422784_at	**Krt6a**	**1.00000**	**0.0000**	**0.0005**	**0.99997**	**0.0002**	**0.0072**
1427700_x_at	**Krt6a**	**1.00000**	**0.0000**	**0.0018**	**0.99923**	0.0099	0.0606
1422588_at	Krt6b	0.53356	0.0206	0.0818	0.53029	0.0259	0.1006
1423952_a_at	**Krt7**	**1.00000**	**0.0000**	**0.0013**	**1.00000**	**0.0001**	**0.0034**
1420647_a_at	**Krt8**	**1.00000**	**0.0002**	**0.0040**	**1.00000**	**0.0001**	**0.0036**
1423691_x_at	**Krt8**	**0.99997**	**0.0004**	**0.0075**	**0.99998**	**0.0002**	**0.0065**
1435989_x_at	**Krt8**	**1.00000**	**0.0003**	**0.0064**	**1.00000**	**0.0001**	**0.0051**
1452166_a_at	*Krt10*	0.17502	0.1615	0.2731	*0.02235*	*0.0044*	*0.0386*
1419230_at	Krt12	0.52232	0.0537	0.1462	0.51138	0.0213	0.0911
1419231_s_at	Krt12	0.51139	0.3999	0.4347	0.50808	0.2976	0.3459
1422454_at	**Krt13**	**1.00000**	**0.0000**	**0.0007**	**1.00000**	**0.0001**	**0.0051**
1437344_x_at	Krt13	0.50704	0.0247	0.0913	0.50506	0.5159	0.4493
1441863_x_at	**Krt13**	**1.00000**	**0.0000**	**0.0012**	1.00000	0.0065	0.0484
1423935_x_at	**Krt14**	**1.00000**	**0.0001**	**0.0024**	0.07911	0.8626	0.5650
1455573_at	Krt14	0.50210	0.1084	0.2188	0.50192	0.0954	0.1978
1460347_at	**Krt14**	**1.00000**	**0.0001**	**0.0022**	0.05873	0.9688	0.5928
1422667_at	**Krt15**	**1.00000**	**0.0000**	**0.0000**	**0.97034**	**0.0316**	0.1121
1448932_at	Krt16	0.49919	0.7411	0.5721	0.49799	0.6516	0.5005
1420193_at	Krt17	0.50183	0.9514	0.6310	0.49543	0.2494	0.3181
1423227_at	***Krt17***	*0.00000*	*0.0543*	0.1473	*0.00000*	*0.0021*	*0.0258*
1448169_at	**Krt18**	**0.99998**	**0.0007**	**0.0097**	**0.99999**	**0.0002**	**0.0066**
1417156_at	**Krt19**	**1.00000**	**0.0000**	**0.0008**	**1.00000**	**0.0001**	**0.0028**
1426284_at	**Krt20**	**1.00000**	**0.0000**	**0.0013**	**1.00000**	**0.0000**	**0.0006**
1418213_at	**Krt23**	**0.99999**	**0.0003**	**0.0059**	**0.99997**	**0.0001**	**0.0028**
1453327_at	Krt24	0.49291	0.4624	0.4641	0.49358	0.5114	0.4475
1418173_at	Krt25	0.49782	0.4264	0.4478	0.50570	0.0270	0.1031
1436160_at	Krt26	0.49957	0.7225	0.5660	0.50066	0.9046	0.5765
1449378_at	Krt27	0.49672	0.5099	0.4856	0.49879	0.5376	0.4579
1430132_at	Krt28	0.49836	0.4118	0.4408	0.50447	0.9682	0.5927
1458354_x_at	Krt28	0.48162	0.0922	0.2000	0.51770	0.5275	0.4541
1421589_at	Krt31	0.50264	0.2633	0.3533	0.50378	0.5817	0.4761
1420728_at	Krt32	0.49806	0.2001	0.3069	0.51330	0.6681	0.5059
1449387_at	Krt33a	0.49860	0.1085	0.2189	0.49872	0.3192	0.3586
1427179_at	Krt33b	0.49789	0.9921	0.6399	0.49425	0.6029	0.4834
1418742_at	Krt34	0.59709	0.0271	0.0967	0.50807	0.8536	0.5624
1420409_at	Krt35	0.49707	0.1695	0.2804	0.49951	0.1533	0.2492
1427751_a_at	Krt36	0.50470	0.9591	0.6330	0.51418	0.0241	0.0968
1437440_at	Krt39	0.52993	0.0874	0.1940	0.51829	0.0163	0.0787
1430208_at	Krt42	0.50579	0.3548	0.4110	0.51001	0.4374	0.4160
1439765_x_at	Krt42	0.50281	0.1225	0.2343	0.51078	0.7373	0.5279
1448457_at	Krt71	0.49284	0.6580	0.5439	0.50034	0.0290	0.1068
1419840_at	Krt72-ps	0.50092	0.7665	0.5796	0.51316	0.1773	0.2685
1436557_at	Krt73	0.48431	0.4368	0.4527	0.50170	0.2193	0.2972
1427378_at	**Krt75**	**1.00000**	**0.0000**	**0.0000**	**1.00000**	**0.0001**	**0.0042**
1453279_x_at	Krt76	0.53986	0.5306	0.4944	0.51653	0.5943	0.4805
1433923_at	Krt77	0.50160	0.6390	0.5369	0.43607	0.1790	0.2699
1438849_at	Krt78	0.54714	0.0002	0.0042	0.51121	0.0072	0.0508
1427352_at	Krt79	0.49871	0.0649	0.1635	0.50595	0.1029	0.2056
1419619_at	Krt80	0.53025	0.2369	0.3345	0.54021	0.1621	0.2563
1427290_at	Krt81	0.51316	0.5903	0.5186	0.52187	0.1735	0.2656
1451551_at	Krt84	0.49913	0.2695	0.3579	0.50030	0.0329	0.1143
1460185_at	Krt85	0.50411	0.3539	0.4105	0.50756	0.0672	0.1655
1427365_at	Krt86	0.50882	0.9997	0.6415	0.50098	0.8896	0.5724
1434535_at	Krt222	0.49923	0.0647	0.1633	0.51431	0.0110	0.0641
1457354_at	Krt222	0.48953	0.8524	0.6045	0.53634	0.6315	0.4929

Key: bold font type denotes those transcripts which significantly increase between E14 and E16; italicized type denotes those transcripts which significantly decrease between E14 and E16; Affy ID represents the transcript ID on the microarray; p.value is the p.value obtained using the functions lmFit and eBayes from the Limma; the q.value is the FDR applied on the p.value. pplr (probability of positive log-ratio) values range from 0 to 1, with values closest to 0 representing the most significantly down-regulated probe sets and values closest to 1, representing the most significantly up-regulated probe sets. Values of 0.5 represent no significant change.

### Quantitative polymerase chain reaction (qPCR) analyses of *Krt15*

Quantitative reverse transcription polymerase chain reaction (qRT-PCR) analyses of aliquots of RNA used in the arrays were undertaken with Roche LightCycler 480 using the programme 95°C for 5 minutes followed by 50 cycles of 95°C for 10 seconds and 60°C for 30 seconds. Signals for *Krt15* were normalised to the average signals of three housekeeping transcripts (*Actb*, *L7* and *Vcl*). Primers were: *Krt15 forward* ggaagagatccgggacaaa and reverse tgtcaatctccaggacaacg; *Actb* forward 5’agccatgtacgtagccatcc3’ and reverse tcacaatgcctgtggtacg3’; *L7 forward* 5’gaggaagaagtttgccctga3’ and reverse 5’ttgtgatagtgctttgccttct3’; and *Vcl* forward 5’cctcaggagcctgacttcc3’ and reverse 5’gccagctcatcagttagtcgt3’. The relative expression between different experimental groups was calculated using REST 2009 Software (Qiagen).

## Results

**Figure 1:**
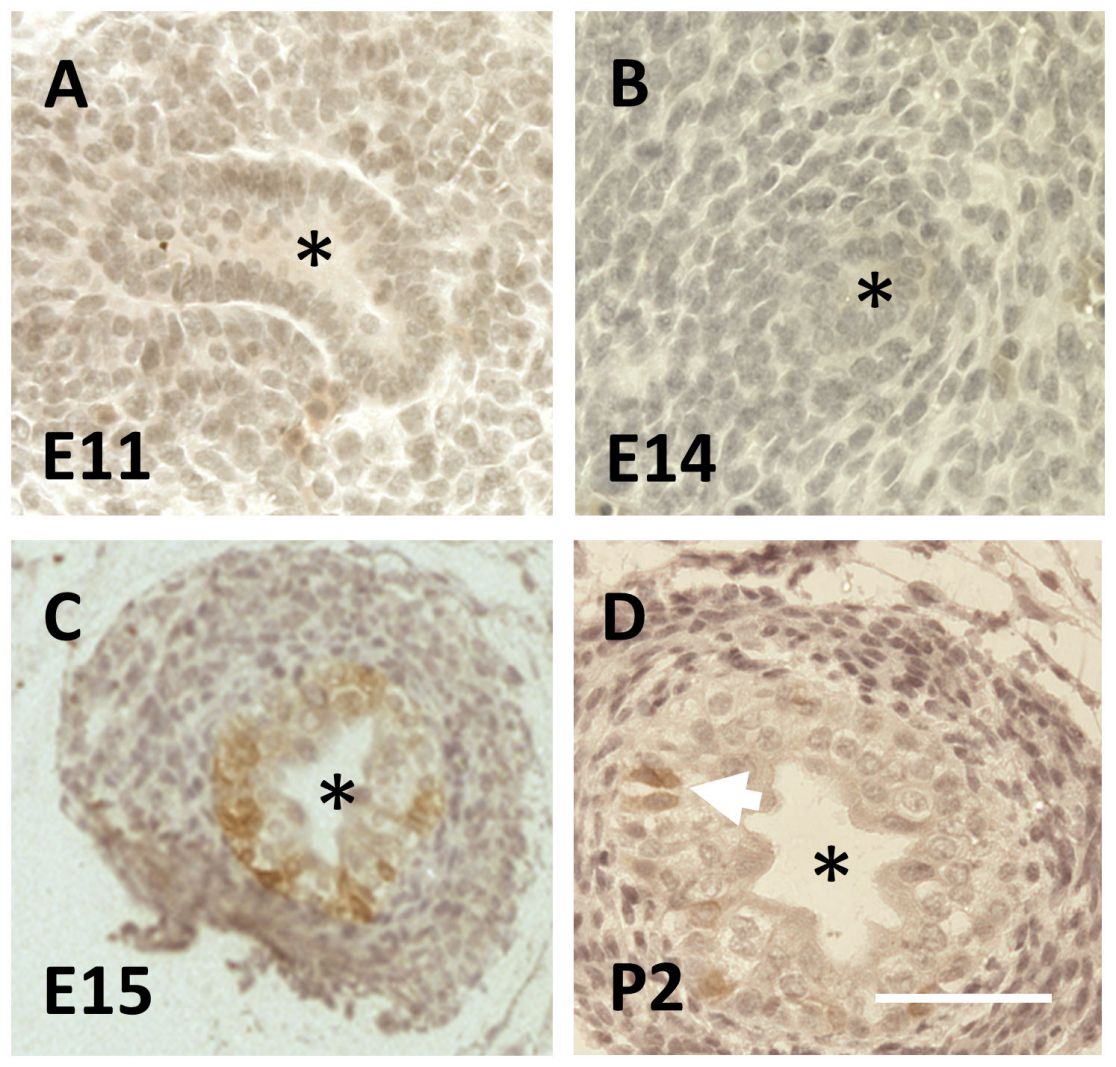
CK15 immunohistochemistry of wild type fetal mouse ureters. In these brightfield images, positive CK15 immunostaining is brown. Nuclei were counterstained blue with haematoxylin. In each image, the asterisk indicates the lumen. **A** and **B**. CK15 was neither detected in the E11 UB (**A**) nor in the monolayered urothelium of the ureteric stalk at E14 (**B**). **C**. A subset of urothelial cells in the multilayered E15 urothelium were positive for CK15. **D**. At postnatal day two (P2), CK15 was detected in a subset of uroltheial cells (two such adjacent cells are indicated by the white arrow). Bar is 200 μm.

The mouse ureter initiates at E10.5 in the form of the UB. Over the next day, the UB enters renal mesenchyme to form the metanephric kidney rudiment. Between E14 and E16, mesenchyme around the extra-renal stalk of the forming ureter becomes induced to form smooth muscle, and the epithelium of the ureteric stalk begins to change from a monolayer to a multilayer, two-three cells thick [7]. Using Affymetrix GeneChip Mouse Genome 430 2.0 microarrays, we found that transcript levels of numerous cytokeratins (detailed in Table 1) altered between these two time points. One of these was *Krt15*, encoding CK15, and its levels increased between E14 and E16 in both proximal (top) and also distal (bottom) sections of the ureter. qPCR analyses confirmed that *Krt15* levels, factored for levels of housekeeping transcripts, increased between E14 and E16 in both the proximal half of the ureteric stalk (average increase, 2.6 fold, n=4, P=0.015), and in the distal half of the organ (average increase, 7.9 fold, n=4, P=0.008). As assessed by brightfield immunohistochemistry, CK15 was not immunodetected in the E11 UB when it was visualised penetrating the renal mesenchyme (Figure 1A), nor was this cytokeratin detected in the ureter stalk up to E14, when its epithelium is a monolayer (Figure 1B). CK15 was, however, detected one day later (E15) when a subset of cells in the now multilayered urothelium were immunostained (Figure 1C). Sparse CK15+ cells remained detectable in the multilayered ureteric urothelium in neonates (Figure 1D). Microarray data were interrogated to determine whether levels of transcripts of other *Krt* genes might be altered between E14 and E16 (Table 1). The following transcripts were significantly upregulated over this two day period: *Krt6a*, *Krt7*, *Krt8*, *Krt13*, *Krt14*, *Krt18*, *Krt19*, *Krt20*, *Krt23* and *Krt75*. In contrast, *Krt4*, *Krt10* and *Krt17* were downregulated over the same timespan. 

**Figure 2:**
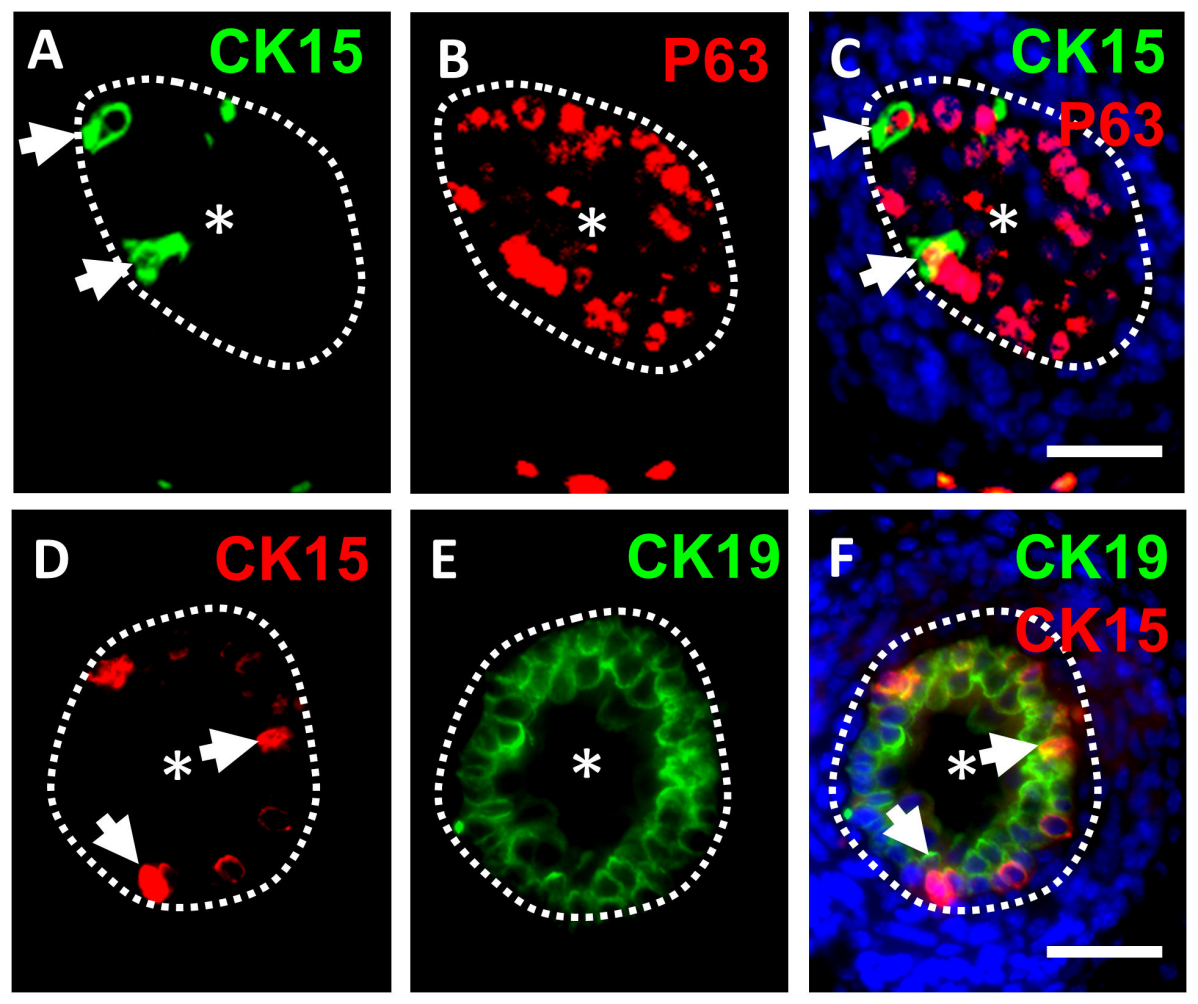
Double immunostaining for CK15/P63 and CK15/CK19. Fluorescence images of cross sections of wild type E17 mouse ureters. In each frame, the asterisk indicates the ureteric lumen and the dotted lines indicate the border between the urothelium and differentiating smooth muscle. **A**-**C**. Double immunostaining for CK15 (green in **A**) and P63 (red in **B**), with the merged images shown in **C** where nuclei are stained blue with DAPI. The same two CK15+ cells are arrowed in A and C; note the presence of P63 in their nuclei. **D**-**F**. Double immunostaining for CK15 (red in **D**) and CK19 (green in **E**), with the merged images shown in **F** where nuclei have been stained blue with DAPI. The same two CK15+ cells are arrowed in **D** and **F**. Note that CK19 has an overlapping but more extensive distribution than CK15. Bars are 100 μm.

**Figure 3:**
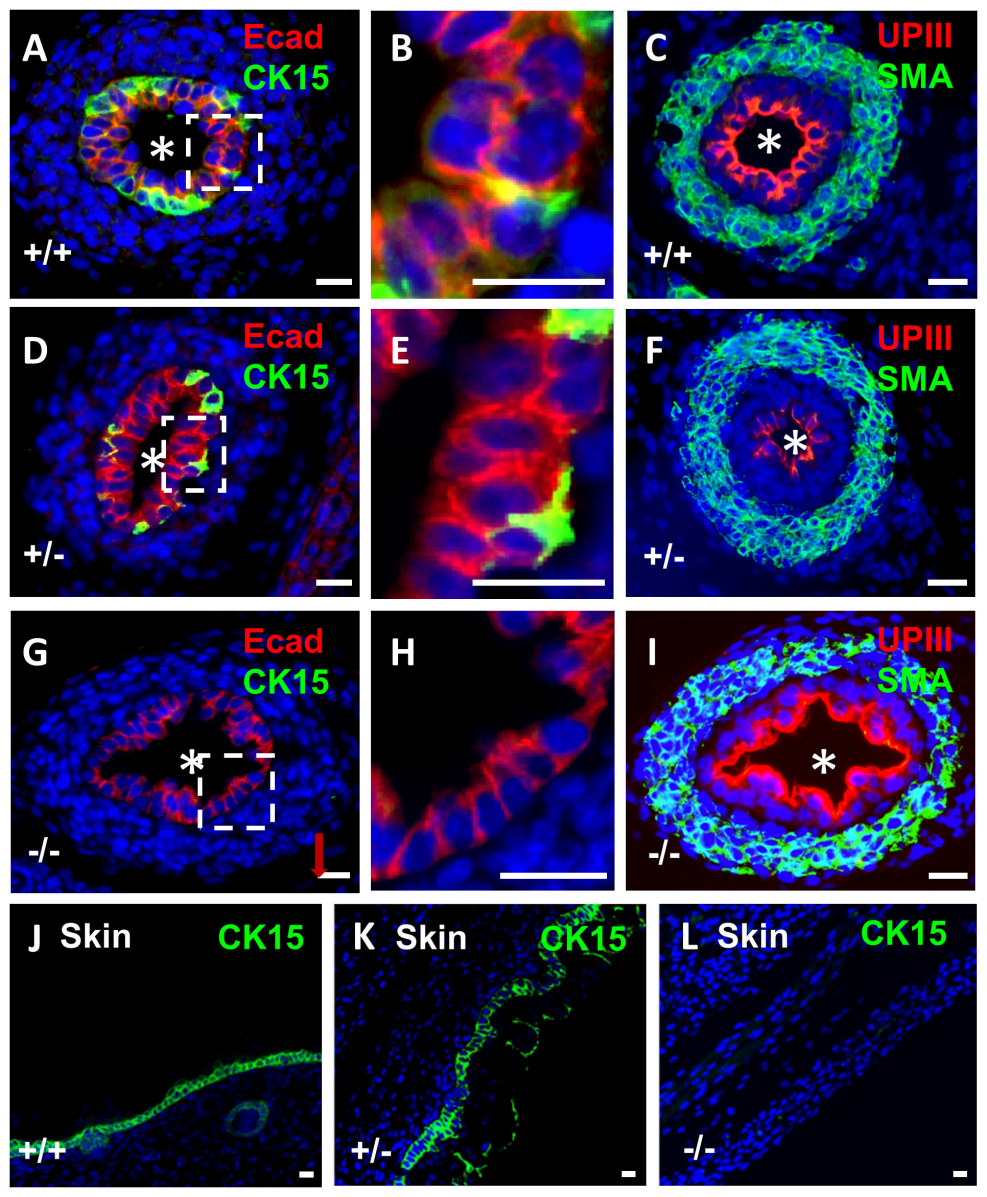
Immunostaining for CK15 in wild type and *p63* mutant embryonic mice. All frames are fluorescence images of E17 organs stained with DAPI (blue nuclei). **A**-**C** and **J**. Tissues from wild type (+/+) mice, **D-F** and K. Tissues from *p63* heterozygous mice. **G**-**I** and **L**. Tissues from homozygous *p63* mutant mice (-/-). **A-I** are ureter cross sections, the asterisks indicating the lumen. The boxed areas around portions of the urothelium in **A**, **D** and **G** have been respectively enlarged and depicted in frames **B**, E and H to show the layer(s) of cells. **J**, K and L are sections through the epidermis. In wild type ureters, CK15 was immunodetected in a subset of basal urothelia (green in A and B) whereas E-cadherin immunoreactivity (Ecad; red in A and B) was detected in all cells of the multilayered urothelium. In wild type ureters, uroplakin III (UPIII; red in **C**) was detected in the most superficial urothelial layer and α-smooth muscle actin (SMA; green in **C**) was immunodetected in the ureteric wall. In *p63* heterozygous mice (**D**-**F**), immunolocalisation patterns for CK15, E-cadherin, uroplakin III and α-smooth muscle actin were similar to wild types. The ureteric urothelium of homozygous *p63* mutant mice (**G**) contained abnormal sections with a monolayer of cells (**G** and **H**) and CK15 was not detected in these organs (**G** and **H**). In contrast to CK15, prominent uroplakin III immunostaining and α-smooth muscle actin were immunodetected in *p63* homozygous ureters. CK15 was immonodetected (green) in wild type (**J**) and *p63* heterozygous (**K**) epidermis. CK15 was not immunodetectable in homozygous *p63* mutant epidermis (**L**). Bars are 20 μm.

To define the location of CK15+ cells within the ureteric urothelium, we undertook fluorescence microscopy using double immunostaining for this cytokeratin and several other proteins, with a focus on E17 ureters, when urothelial multilayering is well-established (Figures 2 and 3). CK15+ cells were only found in the basal-most layer of the ureteric urothelium, where they represented a small subset of cells (Figure 2A, C, D and F). P63, a transcriptional molecule previously implicated in maintenance of the fetal urinary bladder epithelium [24], was immunodetected in nuclei of the majority of basal layer urothelial cells, and CK15+ cells were P63+ (Figure 2B and C). P63 was also detected in some more superficially located urothelial cells but these did not immunostain for CK15 (Figure 2C). As noted above, transcripts for *Krt19*, which encode the acidic cytokeratin CK19, were upregulated during ureter maturation along with those encoding the acidic cytokeratin CK15. In contrast with CK15, however, CK19 protein was more widely detected throughout the multilayered urothelium (Figure 2E and F). 

**Figure 4:**
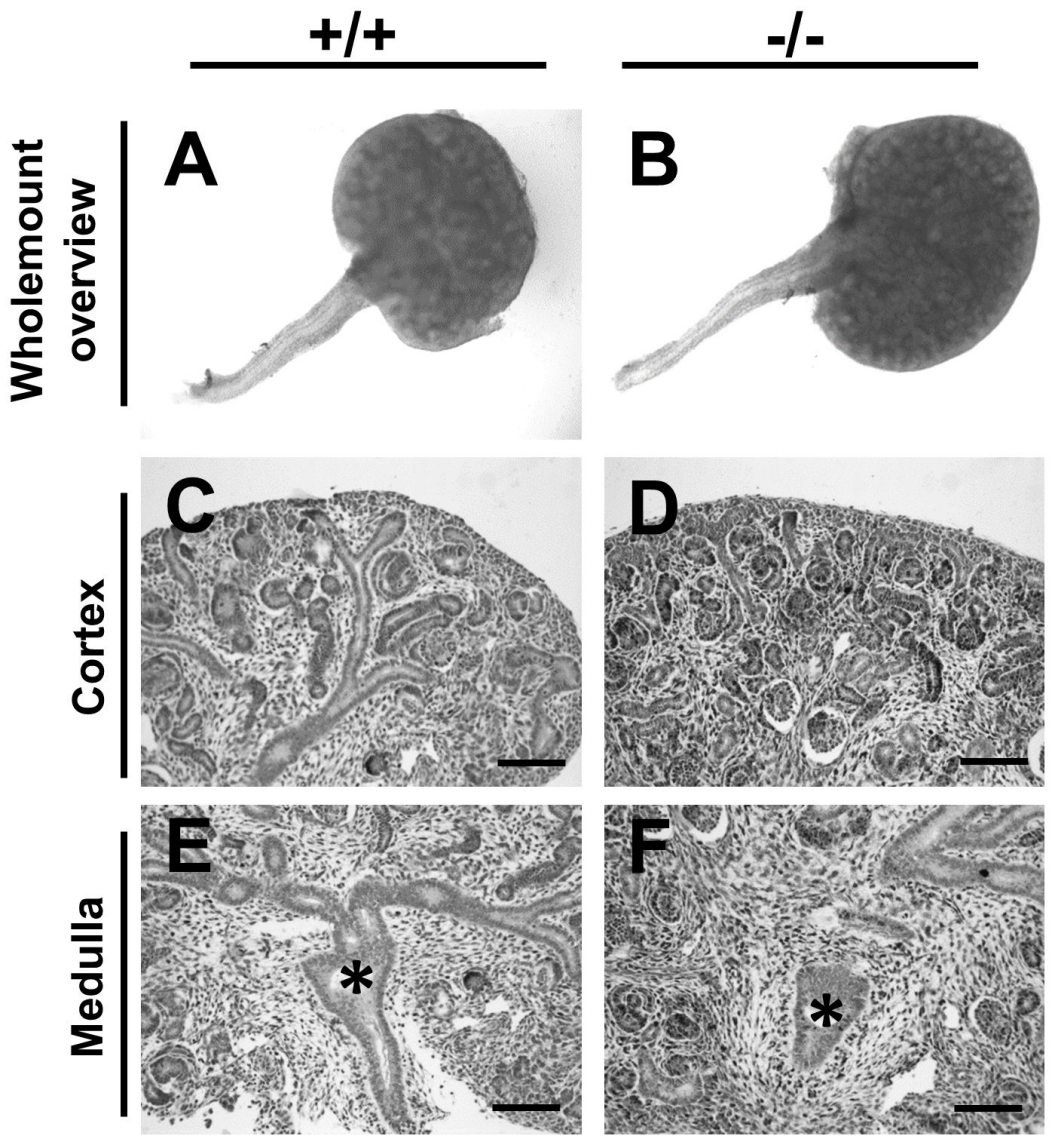
Fetal kidneys and ureters of wild type and *p63* mutant mice. All frames are from E14 organs. **A**, **C** and **E** are from wild type mice (-/-) and B, D and **F** are from *p63* homozygous mutant mice (-/-). **A** and **B**. Whole mount images showing similar gross appearance of renal tracts in the two genotypes. **C**-**F**. Histology sections of the cortex (**C** and **D**) and medulla (**E** and **F**) of the metanephric kidney, with nuclei stained with haematoxylin. Both show several layers of forming nephrons in the cortex and a normal-looking nascent kidney pelvis (asterisks) in the medulla. Bars are 100 μm.

**Figure 5:**
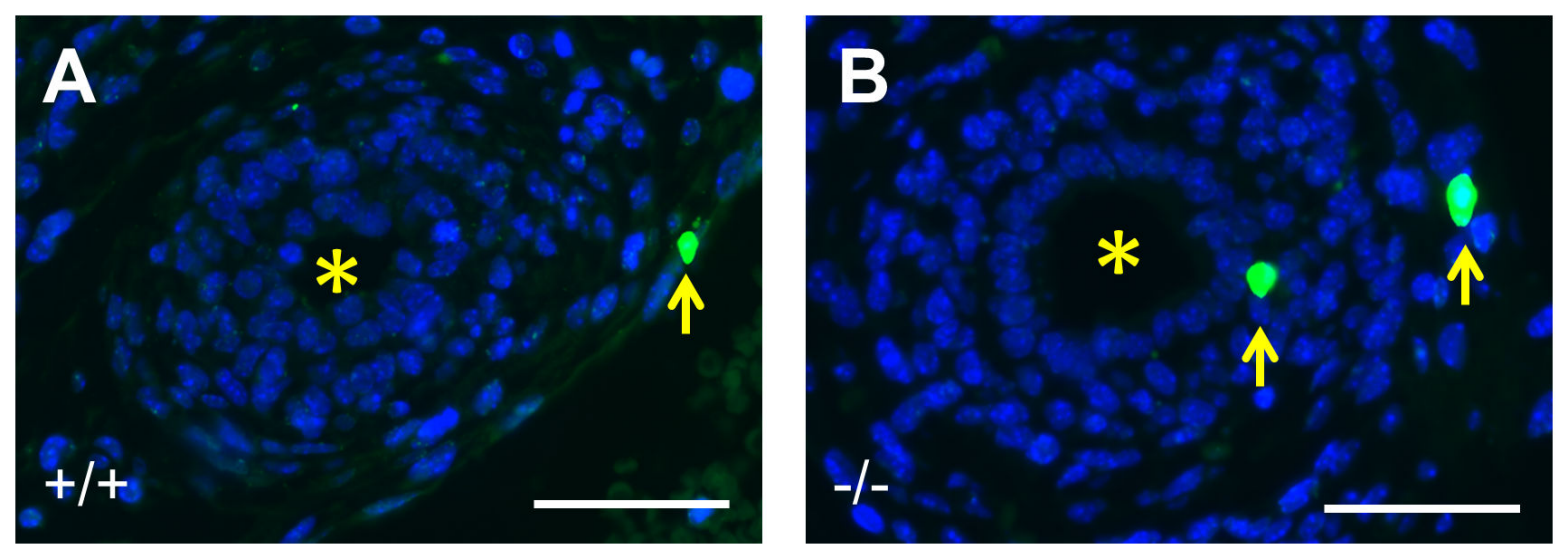
TUNEL labeling of ureters. Transverse sections through ureters with all nuclei stained blue with DAPI; apoptotic nulei are green. The asterisk in each image indicates the ureteric lumen. **A**. Wild type E16 ureter shows no apoptosis in the double-layered epithelium or in adjacent differentiating smooth muscle. There is a single apoptotic nucleus (arrow) in the nearby peritoneal mesothelium. **B**. Homozygous *p63* mutant ureter with two apoptotic nuclei; one (indicated by the arrow on the left) is in the stromal layer immediately outside the epithelial monolayer, while the other (indicated by the arrow on the right) is within loose cells just outside the muscle layer. Bars are 50 μm.

E-cadherin, a cell-cell adhesion protein, was detected throughout the urothelium including the subset of basal cells that were CK15+ (Figure 3A and B). As expected [9,11], the urothelial terminal differentiation marker uroplakin III was immunodetected only in the most superficial urothelial layer (Figure 3C); these umbrella cells did not immunostain for CK15 (compare Figure 3C with 3A). Next, having observed that CK15+ cells also stain positively for P63, we explored the possible effects of *p63* null mutation on CK15. Heterozgous *p63* mice were mated and 12 litters collected between E14 and E18. No gross anomalies were detected in null mutant developing metanephric kidneys as compared with wild type and heterozygous littermate organs at E14 (Figure 4A-F) and other fetal ages (data not shown), nor were null mutant ureters affected by gross malformations such as hydroureter or duplication. In embryonic ureters from *p63* heterozygous mice, the pattern of CK15 immunostaining was similar to wild type littermates i.e. a subset of basal cells were positive (compare Figure 3D with 3A). In ureters (n=4) of homozygous mutant mice, however, CK15+ cells were not detected (Figure 3G). In these ureters, which were not grossly malformed, portions of the urothelium contained only one layer of cells compared with uniform multilayering in wild type and heterozygous littermates (compare Figure 3H with B and E). In contrast to the absence of CK15 protein, ureteric urothelia of *p63* homozygous null mutant mice retained immunostaining for both E-cadherin (Figure 3G and H) and uroplakin-III (Figure 3I), and muscle formation was present, as assessed by immunostaining for α-smooth muscle actin (Figure 3I). TUNEL was undertaken to seek programmed cell death. At E16, apoptoic nuclei were rarely seen in transverse sections of wild type (Figure 5A) and heterozygous ureters. In *p63* homozygous mutant littermates, about half the histological sections contained one or more such nuclei and these were located in the stromal/differentiating smooth muscle layers around the urothelial monolayer (Figure 5A). CK15 was immunodectected in the epidermis of wild type and heterozygous *p63* embryos (Figure 3J and K), yet it was not detectable in homozygous *p63* mutant skin (Figure 3L). 

**Figure 6:**
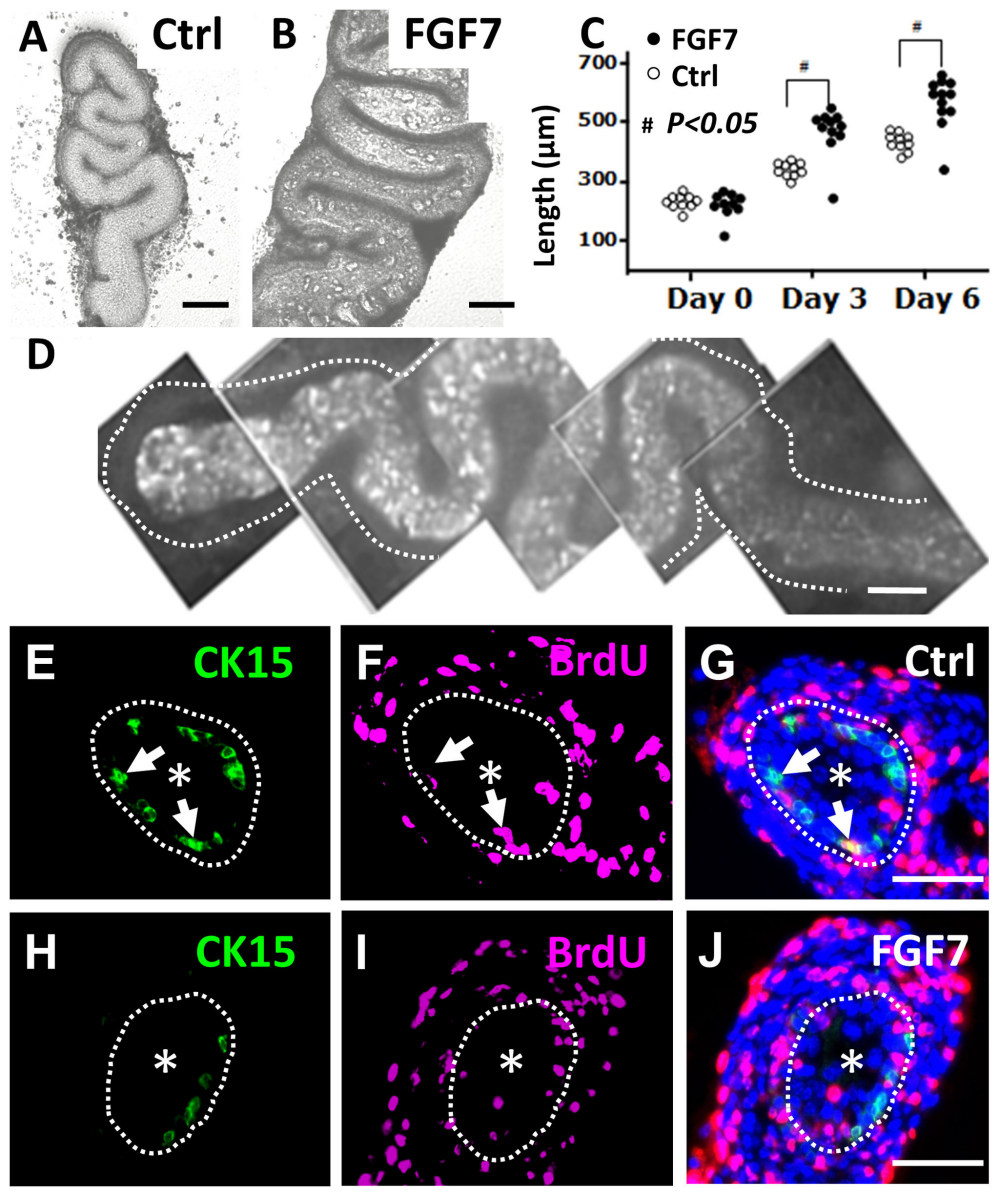
Growth of explanted embryonic ureters. E14 wild type mouse ureters were explanted into organ culture and maintained for up to six days. **A** and **B**. Phase contrast images of whole explants on day six of culture. Ureters cultured in the presence of FG7 (**B**) appeared bulkier than controls (Ctrl in **A**). **C**. Groups of explants cultured with FGF7 were significantly (#; p<0.05, as assessed by t-tests) longer than controls at three and six days of culture (each dot represents one ureter). **D**. Whole mount CK15 immunostaining of ureteric explant on day six of culture showed positive cells in the central, urothelial, core of the organ; the dotted line indicates the perimeter of the ureter and the top/proximal end is on the left of the frame. **E**-**J**. Cross sectional images of explanted ureters fed for six days with control media (**E**-**G**) or media supplemented with FGF7 (**H**-**J**). In each frame, the dotted line marks the border between the urothelium and the surrounding differentiating smooth muscle, and an asterisk has been placed in the lumen. Images E and H demonstrate that CK15+ (green) urothelial cells were present in both experimental groups. Images F and I show BrdU+ cell nuclei in each experimental group. Most of them were in the developing muscle layer but some were present in the urothelium. In the merged images (G and J, where DAPI/blue nuclear staining is also shown), it is apparent the the BrdU+ cells in the urothelium usually do not correspond to the CK15+ cells. In each of the frames **E-G**, the locations of the same two CK15+ cells are arrowed; the upper cell is BrdU- while the lower one is BrdU+. In **H**-**J**, there is a cluster of BrdU+ epithelial nuclei at ‘6-9 o’clock’ and they are separate from CK15+ cells. Bars are 50 μm.

**Figure 7:**
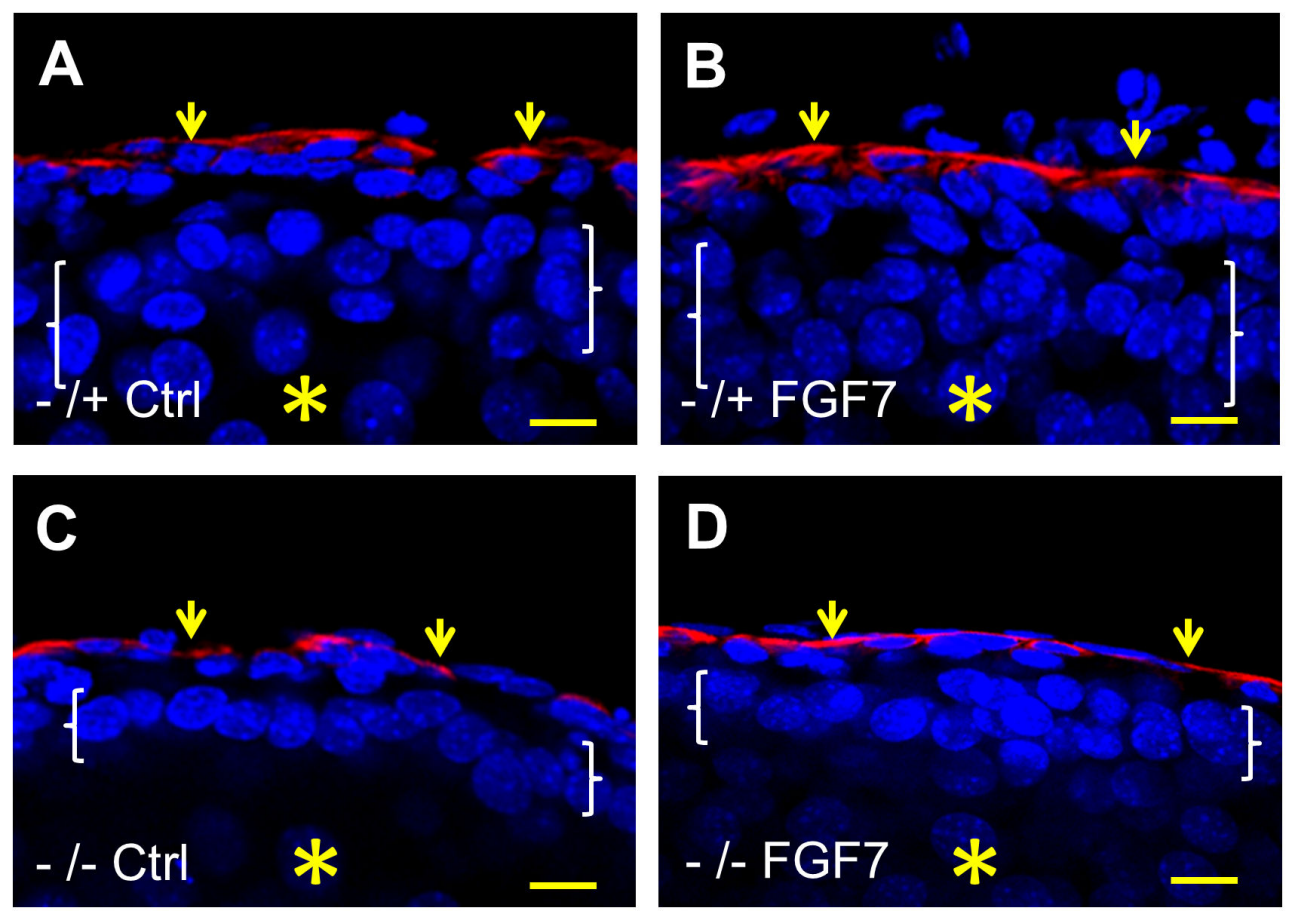
Fluorescence confocal images of embryonic ureter explants. E14 ureters were explanted and maintained for six days in organ culture, without (*Ctrl*) or with addition of FGF7 (*FGF7*). In these longidutinal sections, all nuclei were stained blue with DAPI and the red color indicates positive signal after immunostaining for α-smooth muscle actin, thus marking the muscle layer (small arrows). In each frame the asterisk indicates the (collapsed) lumen and the white brackets span the epithelial layer. Note that the heterozygous *p63* explant displays the expected normal epithelium with 2-4 cell layers (**A**) and that the epithelium is also multilayerd upon exposure to FGF7 (**B**). In contrast, the urothelium in homozygous mutant p63 (-/-) explants generally have only one or two layers, both without (**C**) or with (**D**) FGF7 treatment. Bars are 10 μm.

Explanted E14 wild type mouse ureters elongate and differentiate in serum-free organ culture [7]. FGF7, also known as keratinocyte growth factor, has been shown to enhance growth of the developing ureter *in vivo* [25] and the factor increases proliferation of bladder urothelia [26]. In the current study, explanted wild type organs exposed to FGF7 were significantly longer than control ureters at both three and six days of culture (Figure 6A-C). After six days in culture, whole mount immunostaining revealed CK15+ cells scattered along the length of the ureters fed either control media or media supplemented with FGF7, with positive cells located in the basal layer of the multilayered urothelium (Figure 6D, E and H). Furthermore, in both control and FGF7-supplemented cultures, a subset of epithelial nuclei were found to have incorporated BrdU after two hours exposure to this thymidine analogue; the majority of such BrdU+ cells, however, did not appear to be associated with CK15 immunoreactivity (Figure 6E-J). Given that FGF7 enhanced overall growth of wild type ureters, we hypothesied that application of this growth factor might normalise the lack of multilayering in *p63* null mutant ureters. Exposure of explanted E14 mutant ureters to FGF7 for six days was not, however, associated with an increase in epithelial layering beyond two cells (Figure 7A-D).

**Figure 8:**
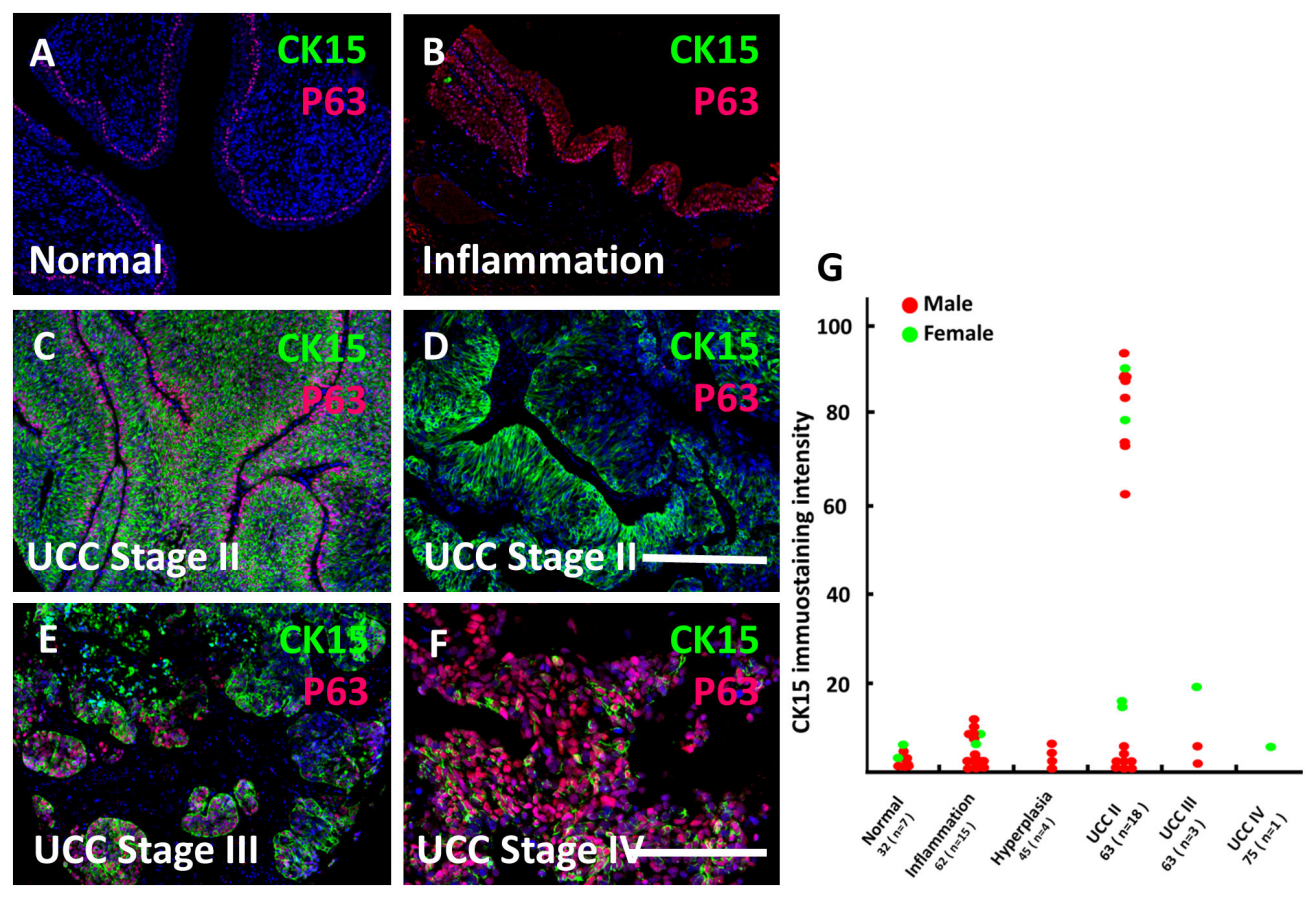
CK15 in human ureteric cancer. **A**-**F**. Fluorescence images of sections of human ureters stained with DAPI (blue) and immunostained for CK15 (green) and P63 (red). Note the lack of significant CK15 immunoreactivity in healthy (**A**) and inflammed (**B**) tissues, whereas P63 was detected in the basal layer of healthy urothelium (**A**) and more extensively within inflamed epithelia (**B**). **C** and **D** depict two different stage II UCC (TCC) samples, each with prominent CK15 immunostaining; there is some heterogeneity of CK15 intensity within the tumour depicted in **D**. CK15 was also detectable in subsets of cells in UCC stage III (E) and IV (**F**) tumours. **G**. Quantification (in arbitrary units) of CK15 immunohistochemical signals across urothelia and tumour sections of individual samples. On the horizontal axis, the first number below each group designation is the average age in years of the set of patients; the number of patient samples (n) is shown in brackets. Bars are 250 μm.

Ureteric cancers are disorders of differentiation and growth. Accordingly, we undertook CK15 immunohistochemistry in a panel of adult human ureter samples which included normal and inflamed tissues as well as epithelial tumours at various stages of invasion. Representative photomicrographs are shown in Figure 8A-F and quantification of CK15 signals from individual samples depicted in Figure 8G. In healthy (Figure 8A) and inflamed (Figure 8B) ureters, and in organs which showed epithelial hyperplasia, CK15 signals were either undetectable or present only at low intensity (Figure 8G). Stage II urothelial cell carcinomas (UCCs) also called transitional cell carcinomas (TCCs), have invaded the lamina propria and have entered the muscle layer. In around half of those assessed, CK15 immunostaining was intense in the majority of tumour cells (Figure 8C and D), an observation which held in tumours resected from both male and female patients (Figure 8G). Although scattered cells in stage III and IV UCCs (i.e. those which had grown through the muscle layer and/or beyond) clearly contained CK15+ cells (Figure 8E and F), the overall immunostaining intensities across these tumours were similar to those measured in non-tumour tissues (Figure 8G). It should be noted that, compared with stage II UCCs, relatively few stage III and IV tumours were present on the tissue array. The sections were co-immunostained for P63. In healthy ureters, this protein was detected in all cells of the basal urothelial layer (Figure 8A), and all epithelial cells were positive in hyperplastic lesions (Figure 8B).

**Figure 9:**
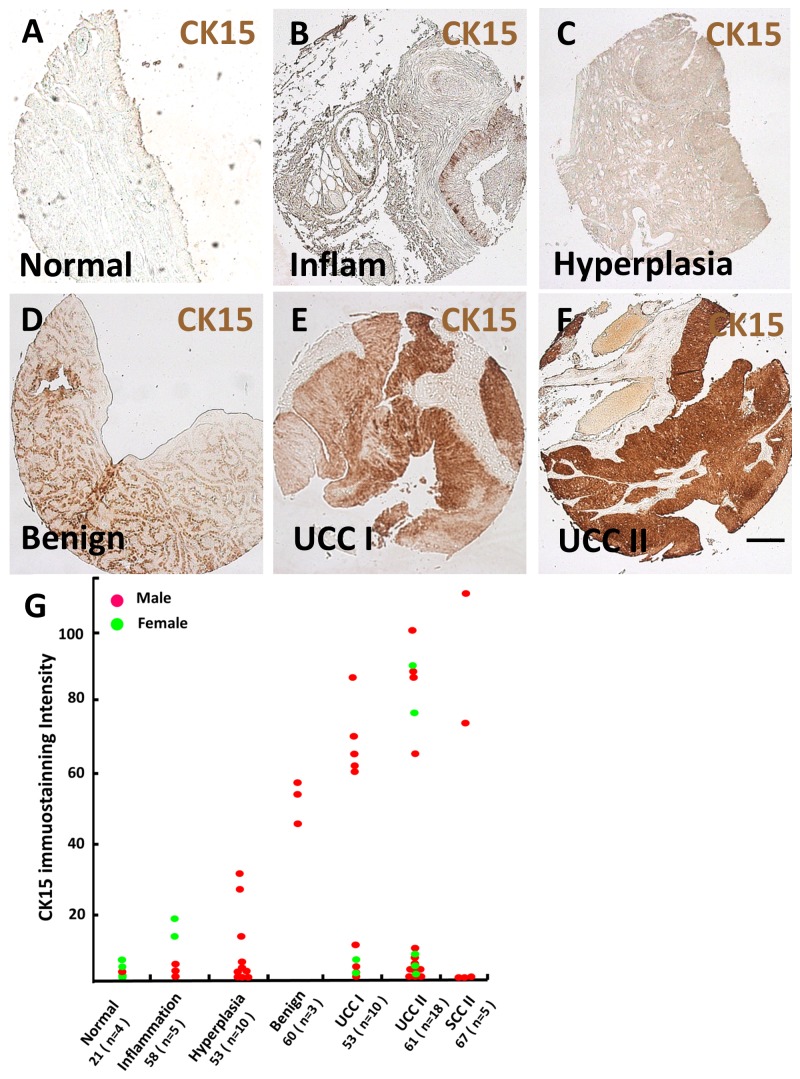
CK15 in human urinary bladder cancer. **A**-**F**. Low power bright field images of histology from adult urinary bladders, from normal healthy (**A**) and pathological (**B**-**F**) tissues, as indicated in the frames (Inflam = inflamed tissue). **G**. Quantification (in arbitrary units) of CK15 immunohistochemical signals across urothelia and tumour sections of individual samples. On the horizontal axis, the first number below each group designation is the average age in years of the set of patients; the number of patient samples (n) is shown in brackets. SCC = squamous cell carcinoma of the bladder. Bar is 200 μm.

Next, bladder tissue samples were immunostained for CK15 (Figure 9). Normal bladder urothelia displayed weak signals (Figure 9A and G) but subsets of pathological samples (Figure 9B-G) displayed increasing CK15 immunostaining as follows: inflammation<hyperplasia<benign tumour (papilloma)<stage I UCC=stage II UCC. Stage III-IV UCCs were not available for study. CK15 immunostaining intensity in two out of five squamous cell carcinomas (all stage II) was prominent (Figure 9G). 

## Discussion

*Krt15* transcripts and/or CK15 protein exist in epidermal [12-16], oesophageal [27] and uterine cervical [28] epithelia and in corneal limbal cells [29]. This study found that *Krt15* transcripts and CK15 protein were upregulated as the ureteric epithelium became multilayered. The basic keratin CK5 may partner CK15 within skin [30] and human *KRT5* mutations cause epidermolysis bullosa simplex. Urethral stenosis with secondary urinary tract obstruction can occur in epidermolysis bullosa [31] but primary ureteric disease has not been reported in individuals with *KRT5* mutations. Moreover, in our mouse study, *Krt5* transcripts did not significantly increase between E14 and E16. *Krt6a*, *Krt7* and *Krt8* transcripts were, however, upregulated along with *Krt15* and so the basic/neutral cytokeratins they encode might be interaction partners for the acidic CK15. Transcripts encoding other other acidic cytokeratins (*Krt13*, *Krt14*, *Krt18, Krt19* and *Krt20*) also increased as the ureter matured. Immunohistochemistry for CK19 showed that it was present in all urothelial layers while CK15 was confined to a subset of basal cells. *Kr4* and *Krt17* RNA levels decreased between E14 and E16, further indicating the complexity of keratin transcript changes during ureter differentiation.

P63 is essential for epidermal stratification [18,19] and is implicated in skin tomourigenesis [32]. To our knowledge, P63 localisation during ureter development and the ureteric phenotype of mutant *p63* mice had not been reported previously. In wild type mice, P63 immunolocalised in most basal cells within the multilayered fetal ureteric urothelium, a zone where CK15+ cells co-expressed P63. In homozygous *p63* mutant ureters, portions of the urothelium were monolayered compared with uniform multilayering in wild type littermates. Furthermore, *p63* homozygous mutant ureters lacked CK15+ cells. *p63* is expressed in urinary bladder epithelia where, in the absence of the ΔNP63 isoform, the urothelium is monolayered [24,33]. Lineage tracing in mice demonstrates that *ΔNp63-*expressing precursor cells can give rise to uroplakin-expressing umbrella cells within the baldder [34]. Nevertheless, P63 may not be essential for umbrella cell differentiation because uroplakin is expressed in *p63* ^*-/-*^ bladder epithelia [33,35]. The current study extends these observations by discovering the *p63* null mutant ureters lack normal multilayering yet uroplakin-III expression is maintained. Bladder dysmorphogenesis in homozygous *p63* mutant mice resembles human bladder exstrophy and mutations in the *P63* promoter have been associated with this disease [36]. Our study suggests that such individuals may also have ureteric urothelial differentiation defects despite lacking gross ureteric malformations. 

Thus, the presence of CK15 within the ureter depends on P63. Perhaps this is a direct, transcriptional, effect. Notably, CK15 was immunodetected in epidermis of wild type and heterozygous *p63* embryos but was absent in homozygous *p63* mutant skin. Kouwenhoven et al [37] undertook genome-wide DNA-binding profiling after chromatin immunoprecipitation in primary, non mutant, human keratinocytes. Deep sequencing identified potential P63 target genes and regulatory elements. Interrogating these analyses (deposited in NCBI's Gene Expression Omnibus and accessible through GEO Series accession number GSE17611), one such target was a DNA sequence 1500 base pairs upstream of the *KRT15* promoter. In future, such an experimental strategy could be applied to confirm whether *KRT15* is an *in vivo* target of P63 in ureteric urothelia. Alternatively, the lack of CK15+ cells in *p63* null ureters might be explained by deletion of CK15+ cells. Indeed, apoptosis is upregulated in mutant *p63* embryonic bladders [24]. Accordingly, we sought apoptosis in histology sections of E16 ureters at which stage apoptotic nuclei were noted in the stromal/differentiating smooth muscle layers but not in the mutant urothelium itself. This observation does not exclude the possibility of deletion of epithelial precursors at an earlier stage. 

The above observations regarding the relation of CK15+ cells to urothelial multilayering suggests that they may be a proliferative population. However, in wild type embryonic ureteric explants, both in basal conditions and when linear growth was accelerated by exogenous FGF7, only a minority of CK15+ cells were proliferative. Indeed, many of the cells which had incorporated BrdU after two hour exposure were located in more superficial layers and were CK15 immunonegative. Within the developing ureter, CK15+ cells may be only slowly cyling although it remains possible that their progeny play a stronger role in active growth. Further experiments, such as those which genetically delete *Krt15* expressing urothelial cells and which lineage trace fates of *Krt15*+ cells after urothelial injury will be required to determine the exact roles of CK15. By applying such strategies to the skin, Ito et al [15] concluded that deletion of *Krt15* expressing cells led to loss of hair follicles and that, after epidermal wounding, progeny of *Krt15+* hair follicle bulge cells contributed to a transient epidermal population which migrated to the centre of the wound. 

In the postnatal period, the ureteric urothelium, as well as the bladder urothelium, can undergo malignant transformation. In both locations, the commonest cancer type is the UCC (or TCC) and it accounts for considerable morbidity and mortality [38-40]. In this study, we found that CK15 immunostaining was upregulated versus healthy urothelium in a subset of invasive (stage II) ureteric tumours. In the renal tract as a whole, ureteric and renal pelvis UCCs constitute only around 10% of all tumours of this type, the remainder being in the bladder. Indeed urothelial bladder cancer is the seventh most common cancer in men and the 17th most common cancer in women [41], and there is some evidence that the biology of upper tract and bladder UCCs may differ [42]. Importantly, ureteric and bladder cancers may not be identical regarding thier pathogenesis and bladder urothelium has a different embryonic origin to that found in the ureter (i.e. endodermal versus mesodermal) [2]. Therefore, we immunostained a panel of bladder tissues for CK15. As we observed for ureteric UCCs, a subset of urinary bladder UCCs versus healthy bladder urothelium showed marked upregulation of CK15 immunohistochemical signals. Compared with the ureter, however, CK15 upregulation was apparently not specific for tumours because a more modest upregulation was also detected in a subset of inflamed or hyperplastic bladder urothelial lesions. 

Larger studies should be undertaken to confirm that a subset of ureteric and bladder tumours contain CK15 protein and to explore the relative levels of CK15 and P63 in different grades and types of tumour. However, there already exist reports of *KRT* expression in urothelial cancers, and their results can be compared with our observations. Gabriel et al [43] undertook RNA profiling in bladder cancers and noted that a history of smoking, an established major risk factor for bladder cancer [41], was positively associated with *KRT15* levels in both non-invasive and invasive bladder cancers. Elsamman et al [44] used microarrays to compare transcript profiles of superficial noninvasive with invasive UCCs of the bladder. *KRT15* transcripts were higher in superficial versus invasive tumours. While these two prior studies have reported transcripts encoding CK15 in bladder tumours, neither explored either protein levels or localisation of this cytokeratin nor did they examine ureter as well as bladder samples. Given that a specific skin tumour has been proven to arise from CK15+ epidermal cells [17], by analogy urothelial CK15 positive cells may be found to have a direct role in ureteric and bladder tumour growth. Perhaps CK15 is part of an expression programme that is activated in UCC tomourigenesis and local invasion and, along with other molecules, may enhance epithelial multilayering in such tumours. However, given our observations that only a subset of ureteric and bladder UCCs upregulate CK15, it could at most have a role in only some UCCs. With regard to P63, Choi W et al [45] undertook qPCR in 101 bladder tumours. They reported that all non-muscle-invasive cancers expressed high levels of E-cadherin and *P63* transcripts. A subset of muscle-invasive tumours maintained high levels of expression of *P63*, and these patients had shorter overall survival. Karni-Schmidt et al [33] analysed 202 human bladder carcinomas by immunohistochemistry and concluded that ΔNp63 was associated with an aggressive clinical course and poor prognosis. Thus, in invasive tumours, there is heterogeneity of P63 expression/protein levels. 

## Conclusions

During normal ureteric development, the appearance of CK15 coincides with the formation of a multilayered urothelium. Conversely, the absence of CK15, in the context of *p63* null mutation, is associated with a simplified urothelium. Postnatally, increased CK15 levels sometimes feature in urothelial overgrowth and in local invasion urothelial tumours. CK15 may be a novel marker for a specific type of urinary tract epithelial precursor cell.

## References

[1] Lye CM, Fasano L, Woolf AS (2010) Ureter myogenesis: putting Teashirt into context. J Am Soc Nephrol 21: 24-30. doi:10.1681/ASN.2008111206. PubMed: 19926888. 19926888

[2] Woolf AS, Davies JA (2013) Cell biology of ureter development. J Am Soc Nephrol 24: 19-25. doi:10.1681/ASN.2012020127. PubMed: 23123402. 23123402

[3] Zhao H, Kegg H, Grady S, Truong HT, Robinson ML et al. (2004) Role of fibroblast growth factor receptors 1 and 2 in the ureteric bud. Dev Biol 276: 403-415. doi:10.1016/j.ydbio.2004.09.002. PubMed: 15581874. 15581874 PMC4131686

[4] Michos O, Cebrian C, Hyink D, Grieshammer U, Williams L et al. (2010) Kidney development in the absence of Gdnf and Spry1 requires Fgf10. PLOS Genet 6: e1000809 PubMed: 20084103. 20084103 10.1371/journal.pgen.1000809 PMC2797609

[5] Pitera JE, Woolf AS, Basson MA, Scambler PJ (2012) Sprouty1 haploinsufficiency prevents renal agenesis in a model of Fraser syndrome. J Am Soc Nephrol 23: 1790-1796. doi:10.1681/ASN.2012020146. PubMed: 23064016. 23064016 PMC3482732

[6] Yu J, Carroll TJ, McMahon AP (2002) Sonic hedgehog regulates proliferation and differentiation of mesenchymal cells in the mouse metanephric kidney. Development 129: 5301-5312. PubMed: 12399320. 12399320 10.1242/dev.129.22.5301

[7] Caubit X, Lye CM, Martin E, Coré N, Long DA et al. (2008) Teashirt 3 is necessary for ureteral smooth muscle differentiation downstream of SHH and BMP4. Development 135: 3301-3310. doi:10.1242/dev.022442. PubMed: 18776146. 18776146

[8] Jenkins D, Woolf AS (2007) Uroplakins: new molecular players in the biology of urinary tract malformations. Kidney Int 71: 195-200. doi:10.1038/sj.ki.5002053. PubMed: 17183244. 17183244

[9] Wu XR, Kong XP, Pellicer A, Kreibich G, Sun TT (2009) Uroplakins in urothelial biology, function, and disease. Kidney Int 75: 1153-1165. doi:10.1038/ki.2009.73. PubMed: 19340092. 19340092 PMC3717210

[10] Kong XT, Deng FM, Hu P, Liang FX, Zhou G et al. (2004) Roles of uroplakins in plaque formation, umbrella cell enlargement, and urinary tract diseases. J Cell Biol 167: 1195-1204. doi:10.1083/jcb.200406025. PubMed: 15611339. 15611339 PMC2172608

[11] Jenkins D, Bitner-Glindzicz M, Malcolm S, Hu CC, Allison J et al. (2005) De novo Uroplakin IIIa heterozygous mutations cause human renal adysplasia leading to severe kidney failure. J Am Soc Nephrol 16: 2141-2149. doi:10.1681/ASN.2004090776. PubMed: 15888565. 15888565

[12] Whitbread LA, Powell BC (1998) Expression of the intermediate filament keratin gene, K15, in the basal cell layers of epithelia and the hair follicle. Exp Cell Res 244: 448-459. doi:10.1006/excr.1998.4217. PubMed: 9806795. 9806795

[13] Waseem A, Dogan B, Tidman N, Alam Y, Purkis P et al. (1999) Keratin 15 expression in stratified epithelia: downregulation in activated keratinocytes. J Invest Dermatol 112: 362-369. doi:10.1046/j.1523-1747.1999.00535.x. PubMed: 10084315. 10084315

[14] Liu Y, Lyle S, Yang Z, Cotsarelis G (2003) Keratin 15 promoter targets putative epithelial stem cells in the hair follicle bulge. J Invest Dermatol 121: 963-968. doi:10.1046/j.1523-1747.2003.12600.x. PubMed: 14708593. 14708593

[15] Ito M, Liu Y, Yang Z, Nguyen J, Liang F, Morris RJ, Cotsarelis G (2005) Stem cells in the hair follicle bulge contribute to wound repair but not to homeostasis of the epidermis. Nat Med 11: 1351-1354. doi:10.1038/nm1328. PubMed: 16288281. 16288281

[16] Garza LA, Yang CC, Zhao T, Blatt HB, Lee M et al. (2011) Bald scalp in men with androgenetic alopecia retains hair follicle stem cells but lacks CD200-rich and CD34-positive hair follicle progenitor cells. J Clin Invest 121: 613-622. doi:10.1172/JCI44478. PubMed: 21206086. 21206086 PMC3026732

[17] Wang GY, Wang J, Mancianti ML, Epstein EH Jr (2011) Basal cell carcinomas arise from hair follicle stem cells in Ptch1(+/-) mice. Cancer Cell 19: 114-124. doi:10.1016/j.ccr.2010.11.007. PubMed: 21215705. 21215705 PMC3061401

[18] Yang A, Schweitzer R, Sun D, Kaghad M, Walker N et al. (1999) p63 is essential for regenerative proliferation in limb, craniofacial and epithelial development. Nature 398: 714-718. doi:10.1038/19539. PubMed: 10227294. 10227294

[19] Mills AA, Zheng B, Wang XJ, Vogel H, Roop DR et al. (1999) p63 is a p53 homologue required for limb and epidermal morphogenesis. Nature 398: 708-713. doi:10.1038/19531. PubMed: 10227293. 10227293

[20] Burgu B, Medina Ortiz WE, Pitera JE, Woolf AS et al. (2007) Vascular endothelial growth factor mediates hypoxic stimulated embryonic bladder growth in organ culture. J Urol 177: 1552-1557. doi:10.1016/j.juro.2006.12.011. PubMed: 17382777. 17382777

[21] Long DA, Kolatsi-Joannou M, Price KL, Dessapt-Baradez C, Huang JL et al. (2013) Albuminuria is associated with too few glomeruli and too much testosterone. Kidney Int 83: 1118-1129. doi:10.1038/ki.2013.45. PubMed: 23447063. 23447063 PMC3674403

[22] Gentleman RC, Carey VJ, Bates DM, Bolstad B, Dettling M et al. (2004) Bioconductor: open software development for computational biology and bioinformatics. Genome Biol 5: R80. doi:10.1186/gb-2004-5-10-r80. PubMed: 15461798. 15461798 PMC545600

[23] Pearson RD, Liu X, Sanguinetti G, Milo M, Lawrence ND et al. (2009) puma: a bioconductor package for propagating uncertainty in microarray analysis. BMC Bioinformatics 10: 211. doi:10.1186/1471-2105-10-211. PubMed: 19589155. 19589155 PMC2714555

[24] Cheng W, Jacobs WB, Zhang JJ, Moro A, Park JH et al. (2006) ΔNp63 plays an anti-apoptotic role in ventral bladder development . Development 133: 4783-4792. doi:10.1242/dev.02621. PubMed: 17079275. 17079275

[25] Qiao J, Uzzo R, Obara-Ishihara T, Degenstein L, Fuchs E et al. (1999) FGF-7 modulates ureteric bud growth and nephron number in the developing kidney. Development 126: 547-554. PubMed: 9876183. 9876183 10.1242/dev.126.3.547

[26] Bassuk JA, Cochrane K, Mitchell ME (2003) Induction of urothelial cell proliferation by fibroblast growth factor-7 in RAG1-deficient mice . Adv Exp Med Biol 539: 623-633. PubMed: 15176316. 15176316 10.1007/978-1-4419-8889-8_40

[27] Leube RE, Bader BL, Bosch FX, Zimbelmann R, Achtstaetter T et al. (1988) Molecular characterization and expression of the stratification-related cytokeratins 4 and 15. J Cell Biol 106: 1249-1261. doi:10.1083/jcb.106.4.1249. PubMed: 2452170. 2452170 PMC2114990

[28] Smedts F, Ramaekers F, Leube RE, Keijser K, Link M et al. (1993) Expression of keratins 1, 6, 15, 16, and 20 in normal cervical epithelium, squamous metaplasia, cervical intraepithelial neoplasia, and cervical carcinoma. Am J Pathol 142: 403-412. 7679549 PMC1886732

[29] Figueira EC, Di Girolamo N, Coroneo MT, Wakefield D (2007) The phenotype of limbal epithelial stem cells. Invest Ophthalmol Vis Sci 48: 144-156. doi:10.1167/iovs.06-0346. PubMed: 17197527. 17197527

[30] Peters B, Kirfel J, Büssow H, Vidal M, Magin TM (2001) Complete cytolysis and neonatal lethality in keratin 5 knockout mice reveal its fundamental role in skin integrity and in epidermolysis bullosa simplex. Mol Cell Biol 12: 1775-1789. doi:10.1091/mbc.12.6.1775. PubMed: 11408584. PMC37340 11408584

[31] Glazier DB, Zaontz MR (1998) Epidermolysis bullosa: a review of the associated urological complications. J Urol 159: 2122-2125. doi:10.1016/S0022-5347(01)63291-9. PubMed: 9598555. 9598555

[32] Keyes WM, Pecoraro M, Aranda V, Vernersson-Lindahl E, Li W et al. (2011) ΔNp63α is an oncogene that targets chromatin remodeler Lsh to drive skin stem cell proliferation and tomourigenesis. Cell Stem Cell 8: 164-176. doi:10.1016/j.stem.2010.12.009. PubMed: 21295273. 21295273 PMC4373450

[33] Karni-Schmidt O, Castillo-Martin M, Shen TH, Gladoun N, Domingo-Domenech J et al. (2011) Distinct expression profiles of p63 variants during urothelial development and bladder cancer progression. Am J Pathol 178: 1350-1360. doi:10.1016/j.ajpath.2010.11.061. PubMed: 21356385. 21356385 PMC3069841

[34] Pignon JC, Grisanzio C, Geng Y, Song J, Shivdasani RA et al. (2013) p63-expressing cells are the stem cells of developing prostate, bladder, and colorectal epithelia. Proc Natl Acad Sci U S A 110: 8105-8110. doi:10.1073/pnas.1221216110. PubMed: 23620512. 23620512 PMC3657776

[35] Signoretti S, Pires MM, Lindauer M, Horner JW, Grisanzio C et al. (2005) p63 regulates commitment to the prostate cell lineage. Proc Natl Acad Sci U S A 102: 11355-11360. doi:10.1073/pnas.0500165102. PubMed: 16051706. 16051706 PMC1183537

[36] Wilkins S, Zhang KW, Mahfuz I, Quantin R, D'Cruz N et al. (2012) Insertion/deletion polymorphisms in the ΔNp63 promoter are a risk factor for bladder exstrophy epispadias complex. PLOS Genet 8: e1003070 PubMed: 23284286. 23284286 10.1371/journal.pgen.1003070 PMC3527294

[37] Kouwenhoven EN, van Heeringen SJ, Tena JJ, Oti M, Dutilh BE et al. (2010) Genome-wide profiling of p63 DNA-binding sites identifies an element that regulates gene expression during limb development in the 7q21 SHFM1 locus. PLoS Genet 6: e1001065. 20808887 10.1371/journal.pgen.1001065 PMC2924305

[38] Lughezzani G, Burger M, Margulis V, Matin SF, Novara G et al. (2012) Prognostic factors in upper urinary tract urothelial carcinomas: a comprehensive review of the current literature. Eur Urol 62: 100-114. doi:10.1016/j.eururo.2012.02.030. PubMed: 22381168. 22381168

[39] Green DA, Rink M, Xylinas E, Matin SF, Stenzl A et al. (2013) Urothelial carcinoma of the bladder and the upper tract: disparate twins. J Urol 189: 1214-1221. doi:10.1016/j.juro.2012.05.079. PubMed: 23023150. 23023150

[40] Kim JJ (2012) Recent advances in treatment of advanced urothelial carcinoma. Curr Urol Rep 13: 147-152. doi:10.1007/s11934-012-0238-0. PubMed: 22367511. 22367511 PMC4009980

[41] Burger M, Catto JW, Dalbagni G, Grossman HB, Herr H et al. (2013) Epidemiology and risk factors of urothelial bladder cancer. Eur Urol 63: 234-241. doi:10.1016/j.eururo.2012.07.033. PubMed: 22877502. 22877502

[42] Yates DR, Catto JW (2013) Distinct patterns and behaviour of urothelial carcinoma with respect to anatomical location: how molecular biomarkers can augment clinico-pathological predictors in upper urinary tract tumours. World J Urol 31: 21-29. doi:10.1007/s00345-012-0946-6. PubMed: 22986906. 22986906

[43] Gabriel U, Li L, Bolenz C, Steidler A, Kränzlin B, Saile M et al. (2012) New insights into the influence of cigarette smoking on urothelial carcinogenesis: smoking-induced gene expression in tomour-free urothelium might discriminate muscle-invasive from nonmuscle-invasive urothelial bladder cancer. Mol Carcinog 51: 907-915. doi:10.1002/mc.20860. PubMed: 21976419. 21976419

[44] Elsamman E, Fukumori T, Ewis AA, Ali N, Kajimoto K et al. (2006) Differences in gene expression between noninvasive and invasive transitional cell carcinoma of the human bladder using complementary deoxyribonucleic acid microarray: preliminary results. Urol Oncol 24: 109-115. doi:10.1016/j.urolonc.2005.07.011. PubMed: 16520272. 16520272

[45] Choi W, Shah JB, Tran M, Svatek R, Marquis L et al. (2012) p63 expression defines a lethal subset of muscle-invasive bladder cancers. PLOS ONE 7: e30206. doi:10.1371/journal.pone.0030206. PubMed: 22253920. 22253920 PMC3254658

